# High-Throughput Phenotyping of Plant Height: Comparing Unmanned Aerial Vehicles and Ground LiDAR Estimates

**DOI:** 10.3389/fpls.2017.02002

**Published:** 2017-11-27

**Authors:** Simon Madec, Fred Baret, Benoît de Solan, Samuel Thomas, Dan Dutartre, Stéphane Jezequel, Matthieu Hemmerlé, Gallian Colombeau, Alexis Comar

**Affiliations:** ^1^INRA, UMR EMMAH, Avignon, France; ^2^ARVALIS – Institut du végétal, Avignon, France; ^3^HIPHEN, Avignon, France

**Keywords:** plant height, high throughput, unmanned aerial vehicles, dense point cloud, LiDAR, phenotyping, broad-sense heritability

## Abstract

The capacity of LiDAR and Unmanned Aerial Vehicles (UAVs) to provide plant height estimates as a high-throughput plant phenotyping trait was explored. An experiment over wheat genotypes conducted under well watered and water stress modalities was conducted. Frequent LiDAR measurements were performed along the growth cycle using a phénomobile unmanned ground vehicle. UAV equipped with a high resolution RGB camera was flying the experiment several times to retrieve the digital surface model from structure from motion techniques. Both techniques provide a 3D dense point cloud from which the plant height can be estimated. Plant height first defined as the *z*-value for which 99.5% of the points of the dense cloud are below. This provides good consistency with manual measurements of plant height (RMSE = 3.5 cm) while minimizing the variability along each microplot. Results show that LiDAR and structure from motion plant height values are always consistent. However, a slight under-estimation is observed for structure from motion techniques, in relation with the coarser spatial resolution of UAV imagery and the limited penetration capacity of structure from motion as compared to LiDAR. Very high heritability values (*H*^2^> 0.90) were found for both techniques when lodging was not present. The dynamics of plant height shows that it carries pertinent information regarding the period and magnitude of the plant stress. Further, the date when the maximum plant height is reached was found to be very heritable (*H*^2^> 0.88) and a good proxy of the flowering stage. Finally, the capacity of plant height as a proxy for total above ground biomass and yield is discussed.

## Introduction

Plant height is recognized as a good proxy of biomass (Yin et al., 2011; Bendig et al., 2014; Ota et al., 2015; Tilly et al., 2015). Stem height that defines plant height appears to be sensitive to the stresses subjected by the crop (Rawson and Evans, 1971). It is also one of the input of models used to evaluate water stress (Blonquist et al., 2009). Plant height is known to make the crop more sensitive to lodging (Berry et al., 2003). Plant height appears thus a highly appealing trait for plant breeders within phenotyping experiments, particularly under natural field conditions. Current methods based on manual evaluation with a ruler on a limited sample size for each microplot are labor intensive, low throughput and prone to errors in the sampling, ruler adjustment, reading and recording the data. Alternative methods have been developed either from LiDAR (Light Detection And Range) often called laser scanning (Hoffmeister et al., 2015), ultrasonic sensors also called sonar (Turner et al., 2007), or from depth camera also called time of flight camera (Chéné et al., 2012; Schima et al., 2016), and finally from RGB high resolution imagery associated with structure from motion algorithms. Depth cameras are limited to close range applications (Schima et al., 2016). Ultrasonic systems are considered as a relatively low-cost solution and user friendly. However, LiDAR measurements have been generally preferred for their increased spatial resolution, higher throughput and independency from air temperature and wind (Tumbo et al., 2002; Escolà et al., 2011; Llorens et al., 2011). LiDAR scanning can be performed from the ground with terrestrial laser scanner). However, terrestrial laser scanners are conical scanners that are well suited for vertically developed objects such as buildings or forests. Their application to crops with limited vertical extent and a canopy volume densely populated by leaves and stems or other organs appears limited (Zhang and Grift, 2012; Bareth et al., 2016): the system needs to be moved over a high number of places for large phenotyping platforms. Further, the several microplots may be seen from different distances and angles with impact on the spatial resolution and associated bias introduced between microplots. It seems therefore preferable to observe crops from near nadir directions.

Several manned or semi-autonomous GPS (Geo-Positioning System) navigated vehicles, have been developed in the recent years where vertically scanning LiDARs have been setup. LiDARs provide a full description of the profile of interception, either with single echo (Lisein et al., 2013; James and Robson, 2014) when the resolution is fine enough, or with full wave form systems (Mallet and Bretar, 2009) or an approximation of it with multi-echo systems (Moras et al., 2010). Because of the penetration of the laser beam into most canopies, nadir looking LiDAR techniques provide at the same time the digital surface model corresponding to the top envelope of the crop (called also crop surface model) and the elevation of the background surface called the digital terrain model. Plant height is then simply computed as the difference between the digital surface model and the digital terrain model. Accuracy on plant height measurement using such LiDAR techniques were reported to be better than a few centimeters (Deery et al., 2014; Virlet et al., 2016). Because of their high accuracy, their independency from the illumination conditions and therefore their high repeatability, these LiDAR based techniques are expected to be more accurate than traditional manual height ruler measurements in the field.

RGB image-based retrieval of crop height remains, however, the most widely used approach (Bendig et al., 2013) because of its low cost and high versatility (Remondino and El-Hakim, 2006). Further, the advances in sensors (smaller, lighter and cheaper, increased resolution and sensitivity) and improvements in computer performances along with advances in algorithms have contributed to the recent success of such techniques (Remondino et al., 2014). The 3D dense point clouds are generated by using a large set of high resolution overlapping images. They are processed using structure from motion algorithms implemented in either commercial software (Smith et al., 2015) such as pix4d^1^, Agisoft photoscan^2^ or in open-source software including mic-mac (MicMac, IGN, France) or Bundler (Snavely et al., 2006). Nevertheless, accurate retrieval of 3D characteristics of the canopy from structure from motion algorithms requires careful completion of the image acquisition that should provide enough view directions for each point of the scene and with crisp high resolution images to identify the tie points used for the 3D reconstruction of the surface (Turner et al., 2014; Smith et al., 2015). Several factors will thus influence the quality and accuracy of the dense point cloud, including flight configuration (altitude, speed, frequency of acquisitions, trajectory design and sensor orientation) camera setting (resolution, field of view, image quality), illumination and wind conditions, the distribution of ground control points as well as the parameters used to run the structure from motion algorithm (Dandois and Ellis, 2013; Remondino et al., 2014).

Because of the spatial resolution of the images used for the structure from motion algorithms and more importantly because of the occlusions observed when a single point is to be seen from two distinct directions, structure from motion algorithms do not penetrate deeply into dense canopies (Lisein et al., 2013; Grenzdörffer, 2014; Ota et al., 2015). Structure from motion technique provides generally a good description of the digital surface model but accessing the digital terrain model is only possible when the ground is clearly visible (Khanna et al., 2015). This is the case for low canopy coverage or for phenotyping platforms where the ground is visible in the alleys and between the plots (Holman et al., 2016). The identification of ground points can be done directly by the photogrammetric software such as Agisoft Photoscan (Geipel et al., 2014). However, this method will depend on the choice of the classification parameters and the type and stage of vegetation. (Khanna et al., 2015) used the green index (Gitelson, 2004) and applied the Otsu automatic thresholding method (Otsu, 1979) over green crops. For senescent vegetation this approach will not provide good results because of confusions between senescent crop and bare soil. Therefore, the generation of the digital terrain model from the dense point cloud appears to be still a challenge in many situations. The problem could be solved by using a digital terrain model derived from an independent source of information (Bendig et al., 2014; Geipel et al., 2014; Grenzdörffer, 2014), assuming that the digital terrain model does not vary significantly during the growing season.

The objective of this study is to develop a methodology for estimating plant height of wheat crops from RGB camera aboard UAV or LiDAR aboard a phénomobile (fully automatic rover) in the context of high-throughput field phenotyping. For this purpose, a comprehensive experiment was setup where the field phenotyping platform was sampled several times during the growing season with the UAV and the phénomobile. A definition of the plant height is first provided from the dense point cloud derived from the LiDAR that will constitute the reference. The UAV derived plant height based on the structure from motion algorithm will then be compared with the LiDAR reference plant height, with emphasis on the way the digital terrain model is computed. The flowering date of wheat was estimated from the dynamics of plant height. Finally, the broad-sense heritability of plant height and its correlation with yield and biomass were evaluated.

## Materials and Methods

### Study Area

**FIGURE 1:**
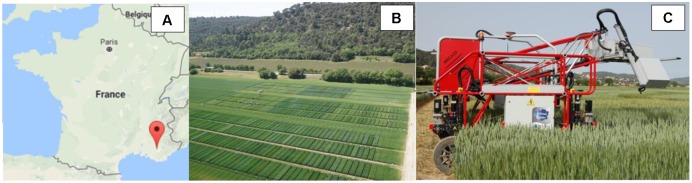
**(A)** Localization of the platform in France; **(B)** Aerial view of the experimental field; **(C)** and the Phénomobile rover robot on which the LiDARs are fixed.

The field phenotyping platform (**Figure 1B**) is located in Gréoux les Bains (France, 43.7° latitude North, 5.8° longitude East, **Figure 1A**). The platform is approximately 200 m by 250 m size and is mainly flat with a 1 m maximum elevation difference. Wheat was sown on October the 29th 2015 with a row spacing of 17.5 cm and a seed density of 300 seeds⋅m^-2^. It was harvested on the 6th July 2016. A total of 1173 microplots of 1.9 m width (11 rows) by 10 m long was considered, each of them corresponding to a given genotype among a total of 550 genotypes grown under contrasted irrigation modalities: about half of the platform was irrigated (WW) while the other part was subjected to water stress (WS modality). A moderate water stress took place in the 2015–2016 season. The cumulated water deficit was 126 mm for the WS modality and 18 mm for the WW modality. A subset of 19 contrasting genotypes was considered here to evaluate the plant height heritability. Each of those genotypes were replicated three times over the WW and WS modalities organized in an alpha plan experimental design.

### Plant Height, Biomass and Flowering Stage Ground Measurements

Plant height was manually measured on 12 microplots: on each microplot, the average of 20 height measurements was calculated; each individual sample measurement corresponds to the highest point of the representative plant within an area of 30 cm radius, corresponding either to leaf or to an ear.

The above ground biomass was measured over three segments of 2 m length by two adjacent rows. The first two rows located at the border of the microplots were not considered in the sampling to minimize border effects. The samples were weighed fresh, and a subsample of around 30 plants taken to measure the water content by weighing it fresh and drying it in an oven for 24 h at 80°C. Around stage Zadoks 26, 6 microplots were sampled. At the stage Zadoks 32, 54 microplots were sampled, corresponding to one replicate of 27 genotypes both in WW and WS modalities. Finally at the flowering stage (Zadoks 50), 80 microplots were sampled corresponding to one replicate of 40 genotypes grown under the two irrigation modalities. However, due to measurement errors, the biomass measurement one microplot was missing. The invasive measurements were taken within less than 4 days from the closest LiDAR survey.

The yield of all the microplots corresponding to 19 genotypes times the three replicates in the two irrigation modalities was measured during the harvest: the weight of harvested grain was divided by the microplot area and the grain fresh weight was normalized to 12% relative moisture.

The flowering date was eventually scored visually every 3 days on one replicate for 19 genotypes grown under both irrigation modalities. The usual scoring system was used: flowering stage corresponds to the date when 50% of the ears have their stamina visible.

### LIDAR Reference Measurements

#### The LiDAR on the Phénomobile

The phénomobile, a ground-based high-throughput phenotyping robot rover is equipped with a measurement head (**Figure 1C**) that is maintained automatically at a constant distance from the top of the canopy. The system steps over the microplots with a maximum 1.35 m clearance and an adjustable width of 2 m ± 0.5 m. The phénomobile automatically follows a predefined trajectory in the experimental field using a centimetric accuracy real time kinetics GPS and accelerometers. The measurement head is equipped with several instruments including two LMS400 LiDARs (SICK, Germany) operating at 650 nm and scanning downward with ±35° zenith angle in a direction perpendicular to the rows at a frequency of 290 scans per second (Lefsky et al., 2002). The two LiDARs allow getting denser sampling of the scene. As the platform moves forward (**Figure 1C**) at a speed of 0.3 m⋅s^-1^ as recorded with the GPS information, the distance between two consecutive scans of a LiDAR along the row direction is around 1 mm. Measurements are taken every 0.2° along the scanning direction. The size of the footprint will depend on the distance to the sensor that varies from 2.4 mm × 5 mm at 0.7 m minimum measuring distance up to 10.5 mm × 5 mm at 3 m maximum measuring distance. The distance between the sensor and the target is measured from the phase shift principle (Neckar and Adamek, 2011). The intensity of the reflected signal and the distance are recorded at the same time. When the target in the LiDAR footprint is not horizontal or made of elements placed at several heights, the distance and the intensity computed by the LiDAR is approximately the average value over the LiDAR footprint. The nominal error on the distance is 4 mm under our experimental conditions. The scan of one microplot takes about 30 s during which about 3 million points are recorded with associated intensity and x-y-z coordinates. Each plot was sampled 14 times during the entire growth cycle to describe the whole season.

#### Data Processing and Height Definition

**FIGURE 2:**
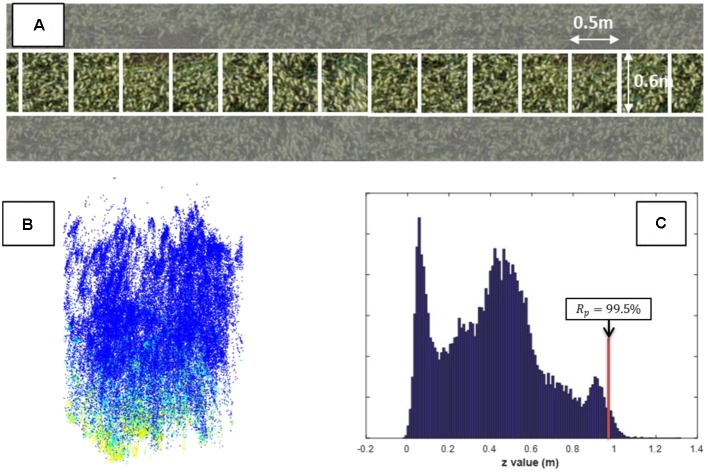
**(A)** UAV-RGB images of one microplot where a 0.6 m × 0.5 m elementary cell is identified; **(B)** The 3D LiDAR points for an elementary cell. Each point colored with the intensity value of the returned signal [from blue (low intensity) to yellow (high intensity)]; **(C)** The corresponding z-distribution of the 3-D points.

A strip of 0.6 m width located in the center of the microplot was extracted from the 3D point cloud (**Figure 2A**). This corresponds roughly to three rows and allows to limit possible border effects while increasing the probability to get points reflected by the soil by limiting the scan angle. Noise from the resulting points where then filtered using the Matlab implementation of the method proposed by Rusu et al. (2008). This process removed about 1% of the points. They were mainly located in the upper and lower part of the regions of interest.

The 0.6 m width strip was further divided into 20 consecutive non-overlapping elementary cells of 0.5 m length where the canopy height was assessed (**Figure 2A**). This allows accounting for possible variation of the digital terrain model if the microplot is not perfectly flat. This cell size was large enough to get a good description of the z profile (**Figure 2C**) including enough points corresponding to the ground level used to define the digital terrain model. The k-means clustering method (Seber, 1984) with two classes was applied to separate the ground from the vegetation from both the distance and the intensity values (**Figure 2B**). The maximum peak in the z-distribution of the resulted non-vegetation points was assigned as the ground level. The distance of the ground was subtracted from the distance of the 3D point cloud for each elementary cell in the microplot resulting into a distribution of the height values. The height of the canopy is then defined as the height value corresponding to a given *R*_p_ of the cumulated height distribution of the vegetation points. The *R*_p_ = 99.5% was selected here to define the vegetation height at the elementary cell level. When considering the later stages where a large heterogeneity of the height is observed at the top layer because of the presence of ears, this corresponds roughly to the area covered by 50 ears for each unit ground area, considering an ear diameter of 1 cm and a typical ear density. The sensitivity of the height to this percentile value will be later discussed in the results section. Finally, the median value of the elementary cells of the microplot was considered as the plant height.

### Plant Height Estimates from the UAV

#### RGB Camera and UAV Flight

A Sony ILCE-6000 digital camera with a 6000 × 4000 pixels sensor was carried by a hexacopter with approximately 20 min autonomy. The camera was fixed on a 2 axes gimbal that maintains the nadir view direction during the flight. The larger dimension of the image was oriented across track to get larger swath. The camera was set to speed priority of 1/1250 s to avoid movement blur. The aperture and ISO were thus automatically adjusted by the camera. The camera was triggered by an intervalometer set at 1Hz frequency that corresponds to the maximum frequency with which RGB images can be recorded on the flash memory card of the camera. The images were recorded in the jpg format. Two different focal lengths were used: 19 and 30 mm with respectively ± 31.0° and ± 21.5° field of view across track. The flight altitude was designed to get around 1 cm GSD for both focal lengths (**Table 1**). Five measurements were completed from tillering to flowering (**Table 1**).

**Table 1:** Characteristics of the five flights completed over the Gréoux experiment in 2016.

Date (DaS)	Illumination conditions	Wind speed (km/h)	Focal length (mm)	Altitude (m)	GSD (cm)	Overlap (%)		σx (cm)	σy (cm)	σz (cm)
									
						along	across			
139	Covered	8	30	75	0.98	90	70	2.4	3.1	5.5
152	Sunny	6	30	75	0.98	90	70	4.5	1.3	3.3
194	Sunny	10	19	50	1.04	94	70	5.1	1.3	3.9
216	Cloudy	7	19	50	1.04	94	70	2.1	2.9	2.8
225	Sunny	5	30	75	0.98	90	70	5.0	2.6	3.9


*σx, σy, σz, correspond to the standard deviation of the localization of the control points used to quantify the geometric accuracy.*

**FIGURE 3:**
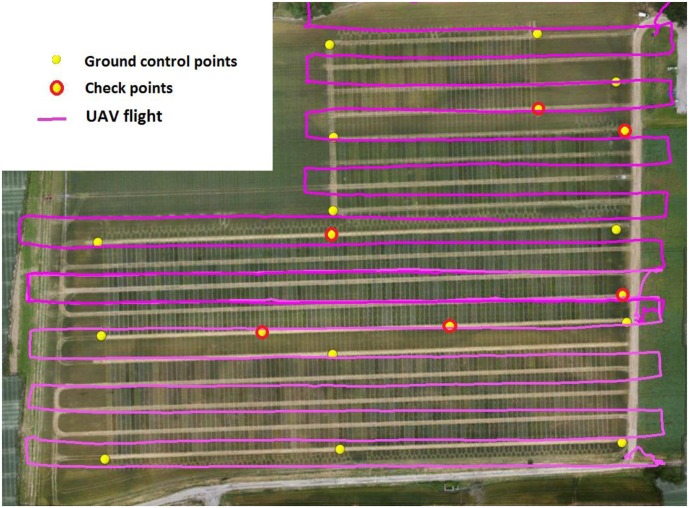
The flight plan with ground control points (yellow circles with red outline) and check points (yellow circles).

The speed of the UAV was set to 2.5 m/s to provide 90 and 94% overlap between images along the track respectively for the 30 mm and 19 mm focal lengths. The distance between tracks was set to 9 and 11.8 m respectively for the 19 and 30 mm focal lengths to provide 70% overlap across track. Two elevations of 10–15 min were necessary to cover the full area of interest. No images were acquired during the UAV stabilization over the waypoints. In addition, images corresponding to the takeoffs and landings were not used. This resulted in about 600 images for each date. The typical flight plan is shown in **Figure 3**.

#### Ground Targets and Georeferencing Accuracy

A total of 19 ground targets were evenly distributed over the platform with fixed position for all the flights. They were made of painted PVC disks of 60 cm diameter where the central 40 cm diameter disk was 20% gray level and was surrounded by a 60% gray level color external crown. These gray levels were selected to avoid saturation and allow automatic target detection on the images. Their location was measured with a real time kinetics GPS device ensuring a 1 cm horizontal and vertical accuracy for every flight. Among the 19 targets, 14 were used in the generation of the dense point cloud (ground control points) while the five additional ones were used to evaluate the accuracy of the geo-referencing (Check Points). The spatial distribution of the targets was designed to get some even coverage of the field considered (**Figure 3**).

#### Generation of the 3D Dense Point Cloud from the RGB Images

The ensemble of RGB images was processed with Agisoft Photoscan Professional (V 1.2.6) software. The first step consists in the image alignment performed using the scale invariant feature transform algorithm (Lowe, 2004). An “on-the-job-calibration” was applied to adjust the camera parameters within the structure from motion process. The application of this method was possible because of the high overlap between images (Turner et al., 2014) and the suitable distribution of the ground control points (James and Robson, 2014; Harwin et al., 2015). The Agisoft software generates in a first step a set of tie points, each point being associated with a projection error. As advised by Agisoft, tie points with a projection error higher than 0.3 ground sample distance were removed. A bundle adjustment is then applied (Granshaw, 1980; Triggs et al., 1999). Further, points with a low reconstruction uncertainty (points, reconstructed from nearby photos with small baseline) were then removed. These points are generally observed for small overlapping fraction between images along with a large view zenith angle resulting in larger ground sample distance. The ground control points used in this process were automatically identified using a custom developed pipeline. The check points were not used in the bundle adjustment, the average accuracy on the check points reported in **Table 1** (σx, σy, and σz) were in agreement with the recommendations from (Vautherin et al., 2016): 1–2 times the ground sample distance in x and y directions, and 2–3 times the ground sample distance in the z direction. The dense point cloud is generated from dense-matching photogrammetry using a moderate depth filtering option and the full image resolution as implemented in Photoscan 1.2.6. This filtering process results in more variable density of points of the dense cloud, the mean density of points in the vegetation part of the study area was 2300 points/m^2^.

#### Derivation of the Digital Terrain Model

Two methods were used to derive the digital terrain model. The first one is simply based on the collection of the coordinates of the points recorded during sowing by the sowing machine equipped with a centimetric accuracy Real Time Kinematic GPS. The second approach is based on the extraction of ground points from the dense point cloud and interpolation between them to generate the digital terrain model. The phenotyping platform (**Figure 3**) was split into 13 m × 13 m cells with a 75% overlapping (50% in both x and y directions). The size of the cell is a compromise between a small one that allows to get of finer description of digital terrain model variations, and a large one that will ensure to get at least few background points from the dense cloud points. Similarly to the LiDAR processing, a k-means clustering (Seber, 1984) with 2 classes is applied using the z-value and the red and green color associated to each point of the dense cloud. This k-means clustering is iterated over the previous background class if the standard deviation in the background class, σ_b_, is lower than 0.14 m. However, if σ_b_ > 0.14 m after the 4th iteration the iteration process is stopped and no background *z*-value is assigned to the considered cell. The σ_b_ > 0.14 m value corresponds approximatively to the background roughness expected over the 13 m × 13 m cell and was defined after several trial and error tests. Then, ground point cloud was filtered using (Rusu et al., 2008) algorithm to regularize the *z*-values over each cell. Finally, a natural neighbor interpolation (Owen, 1992) was applied to compute the z value for each microplot. Note that here the microplot is assumed to be flat.

#### Plant Height Estimation

For each plot, the *z*-values of the dense cloud points were subtracted from the *z*-value of the digital terrain model assigned to the microplot. Finally, the microplot is divided into 20 consecutive non-overlapping elementary cells of 50 cm × 60 cm similarly to what was achieved for the LiDAR data. The median value of plant height corresponding to a given *R*_p_ of the cumulated z distribution is finally computed and considered as the microplot crop plant height. The selection of the value of the *R*_p_ used to define plant height will be discussed later in the Section “Results.”

### Date When the Maximum Plant Height Is Reached

The flowering date appears roughly when the vegetative growth is completed, i.e., when the stems reached their maximum height. This stage could thus be tentatively estimated using the plant height time course. This requires obviously frequent observations as completed in this study with the LiDAR while the plant height monitoring with the UAV was too sparse. As a consequence, only the LiDAR measurements will be used here for estimating the flowering stage. When expressing the time in GDDs the plant height temporal profile can be approximated by a vegetative growth phase, followed by a plateau during the reproductive phase. The plant height corresponding to the plateau was simply defined by the maximum plant height value over the whole cycle. A second-order polynomial regression was used to describe the plant height during the vegetative growth. The vegetative growth period was assumed to start for GDD = 1000°C.day. It was then incrementally extended by including additional observation dates for GDD > 1500°C.day if the corresponding plant height elongation rate does not decrease by more than 60% than that of the previous value. The intersection of the elongation curve with the plateau provides the date when plant height reaches its maximum.

## Results and Discussion

### LiDAR Measurements of Plant Height

**FIGURE 4:**
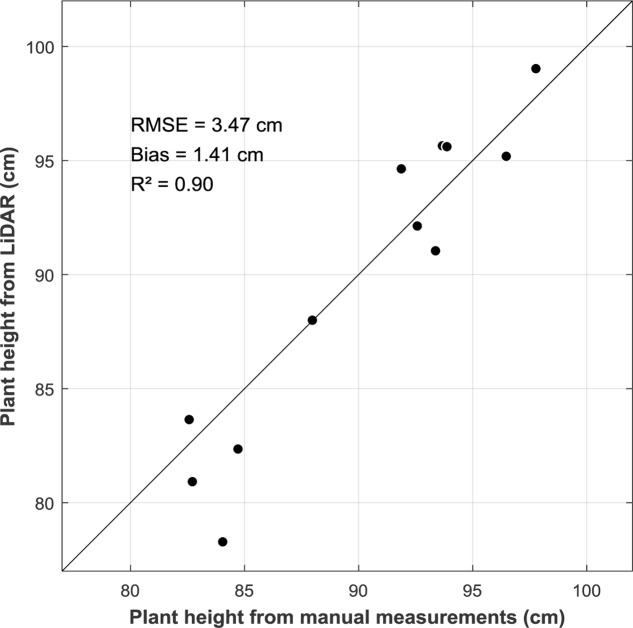
Comparison between plant height derived from LiDAR measurements with plant height measured manually in the field (*n* = 14). Solid line is the 1:1 line.

The LiDAR plant height defined using *R*_p_ = 99.5% were compared with the available manual measurements in the field. Results show a strong agreement with a low RMSE of 3.47 cm and small bias (bias = 1.41 cm) (**Figure 4**).

**FIGURE 5:**
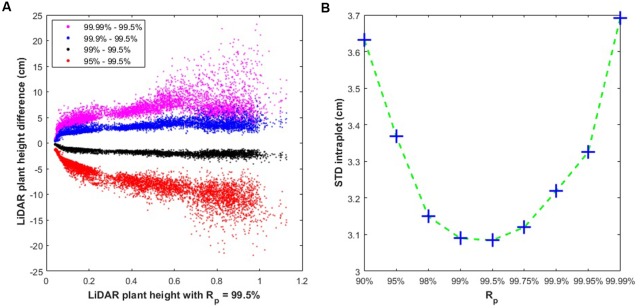
**(A)** Difference in plant height estimates using several *R*_p_ *z*-values (*R*_p_) against estimate using *R*_p_ = 99.5; **(B)** Standard deviation of plant height (STD intraplot, in cm) computed within a microplot between the 20 elementary cells as a function of the *R*_p_ value selected to define plant height.

The impact of the *R*_p_ value on plant height was further investigated using the difference Δ*PH* = *PH*_x_ - *PH*_99.5_ where *PH*_x_ and *PH*_99.5_ represent the plant height values respectively for *R*_p_ = *x%* and *R*_p_ = 99.5%. Results (**Figure 5A**) show that very high values of *R*_p_ = 99.99% increases plant height by more than Δ*PH* = +5 cm in most situations. Conversely, *R*_p_ = 99.0% decreases plant height by more than Δ*PH* = -5 cm. The absolute difference Δ*PH* increases rapidly with plant height for PH < 0.1 (**Figure 5A**). Then, Δ*PH* increases only slightly with plant height (**Figure 5A**), with, however, significant scatter for the larger plant height values and when *R*_p_ is different from the nominal value (*R*_p_ = 99.5%). The variability of plant height across the 20 elementary cells within a microplot (**Figure 5B**) shows that it is minimum for *R*_p_ = 99.5% with STD = 3.1 cm. It increases rapidly either for *R*_p_ < 99.5% or for *R*_p_ > 99.5% although the STD value keeps relatively small (STD < 3.7 cm for *R*_p_ = 99.99% or for *R*_p_ = 90%). The use of the median values computed over the 20 elementary cells provides in addition a better representativeness of the plant height of a microplot. This appears more important at the tillering stage where the plant height variability within a microplot is the largest. These results confirm that *R*_p_ = 99.5% provides an accurate and precise plant height estimation.

### Derivation of the Digital Terrain Model with Structure from Motion Algorithm

The digital terrain model extracted from the dense point cloud for each of the 5 flights were compared. In addition, the digital terrain model generated from the real time kinetics GPS placed on the sowing machine during sowing was also used. A mean altitude value of the ground level for the 1173 microplots was then computed for the 7 digital terrain models. Results show that the correlation between the altitudes computed from all the digital terrain model combinations is always very high with *R*^2^> 0.97 (**Table 2**). This indicates that all the digital terrain models were capturing consistently the general topography of the experimental platform.

**Table 2:** Correlation (*R*^2^, bottom triangle) and RMSE (top triangle) values between the digital terrain models computed over the 1173 microplots for the 5 flights as well as that derived from the real time kinetics GPS on the sowing machine.

R^2^/RMSE (cm)	Sowing	DaS 139^∗^	DaS 152^∗^	DaS 194	DaS 216	DaS 225^∗^
Sowing	-	2.6	7.2	3.4	2.6	6.0
DaS 139^∗^	1.00	-	7.2	4.5	2.9	5.0
DaS 152^∗^	0.96	0.95	-	9.2	7.4	9.8
DaS 194	0.99	0.99	0.95	-	3.8	6.8
DaS 216	0.99	0.99	0.96	0.99	-	6.0
DaS 225^∗^	0.97	0.97	0.91	0.97	0.96	-


*^∗^Indicates that the camera was equipped with the 30 mm focal length instead of the 19 mm one.*

Results show further that the RMSE values are between 2.6 and 6.8 cm (**Table 2**), except for DaS 152 that shows larger values. No clear explanation was found for the degraded performances of DaS 152. However, better consistency seems to be observed when using a shorter focal length (comparison between DaS 194, DaS 216 and Sowing).

### Comparison of Plant Height Derived from Structure from Motion and LiDAR

**FIGURE 6:**
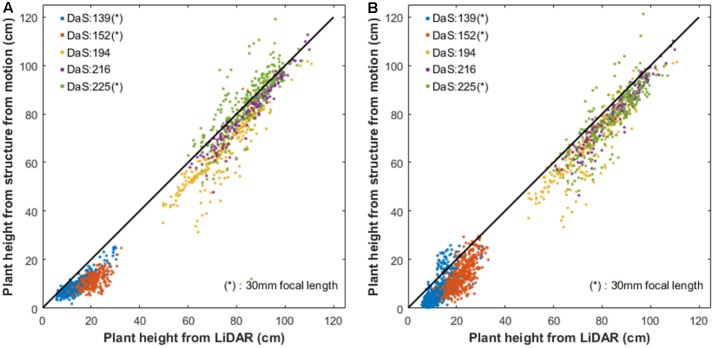
**(A)** Plant Height computed from the background points identified over each date; **(B)** Plant height computed from the digital terrain model derived from the sowing machine. Each color corresponds to a flight date (DaS). ^∗^Indicates that the camera was equipped with the 30 mm focal length instead of the 19 mm one.

The LiDAR was more frequently sampling the platform along the growth season as compared to the UAV flights (**Table 1**). Plant height derived from the LiDAR were thus interpolated to the dates of the UAV flights. However, if the LiDAR acquisition of a microplot differs by more than a week from that of the UAV flight, the corresponding microplot was not considered in the comparison. This resulted in a total of 2076 couples of structure from motion and LiDAR plant height. The plant height from structure from motion was first derived using the same *R*_p_ as that used for the LiDAR (*R*_p_ = 99.5%). Results (**Figure 6**) show that structure from motion plant height are strongly correlated with LiDAR reference plant height across the 5 UAV flights available. This corroborates previous results reported (Bareth et al., 2016; Fraser et al., 2016; Holman et al., 2016). The same level of consistency is observed for plant height derived from a digital terrain model computed from the same dense cloud (*R*^2^ = 0.97, RMSE = 7.7 cm) as compared to using the digital terrain model derived from the sowing (*R*^2^ = 0.98, RMSE = 8.4 cm). The correlations are generally weaker for the early stages due to the limited range of variation of plant height (DaS 139, DaS 152). Further, using the 30 mm camera focal length (DaS 139 DaS 152 DaS 225) tends to decrease the plant height consistency with the reference LiDAR derived plant height as compared to the 19 mm focal length (**Table 3**). The 19 mm focal length increases the disparity in the view configurations which may help the structure from motion algorithm to get more accurate estimates of the z component in the dense cloud as earlier reported (James and Robson, 2014). This result also confirms the ability of Agisoft to model the radial lens distortion of wide field of view lens. However, the calibration of the camera from the bundle adjustment requires an even distribution of a sufficient number ground control points (James and Robson, 2014; Harwin et al., 2015) and a high overlapping between images as done in this study.

**Table 3:** Agreement between LiDAR and structure from motion derived plant height when the digital terrain model used come either from the same dense cloud or from the Sowing.

	Digital terrain model from the dense cloud			Digital terrain model from Sowing		
		
DaS	*R*^2^	RMSE (cm)	Bias (cm)	R^2^	RMSE (cm)	Bias (cm)
139^∗^	0.76	5.0	-4.4	0.50	6.8	-5.6
152^∗^	0.31	9.2	-8.6	0.45	9.0	-9.0
194	0.84	11.0	-9.4	0.80	9.9	-7.7
216	0.92	5.1	-3.9	0.91	6.2	-5.0
225^∗^	0.59	8.7	-0.38	0.63	9.8	-5.4
All	0.97	7.7	-5.1	0.98	8.4	-6.5


*^∗^Indicates that the camera was equipped with the 30 mm focal length instead of the 19 mm one.*

A systematic overestimation of the plant height derived from structure from motion is observed as compared to the reference plant height derived from the LiDAR. This agrees with results from other studies (Grenzdörffer, 2014; Bareth et al., 2016; van der Voort, 2016) who found that structure from motion lacked the ability to reconstruct accurately the top of the canopy. This is partly due to the spatial resolution difference between the LiDAR (3–5 mm) and the RGB camera (10 mm) as compared to the size of the objects at the top of the canopy (on the order of the cm). However, increasing the spatial resolution will lead to more noisy dense cloud with more gaps over vegetated areas as reported by Brocks et al. (2016) and as was experienced also in this study (results not shown for the sake of brevity).

**FIGURE 7:**
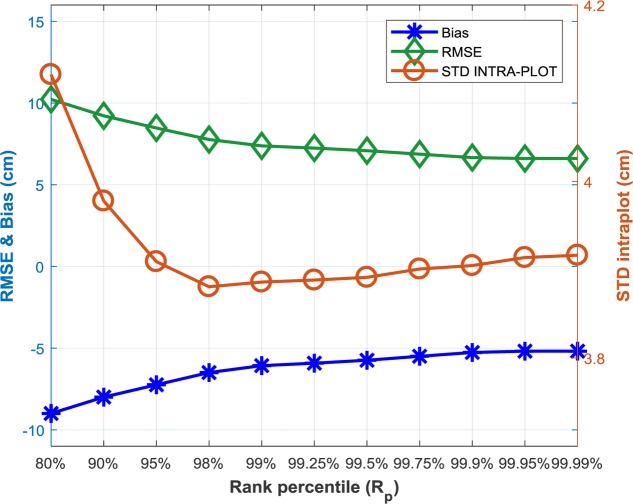
Impact of the rank percentile (*R*_p_) used to defined plant height from the dense cloud derived from structure from motion on RMSE and bias (left *y* axis) and the variability of plant height along the microplot (right *y* axis). The reference plant height used here is that derived from the LiDAR with Rp = 99.5%.

**FIGURE 8:**
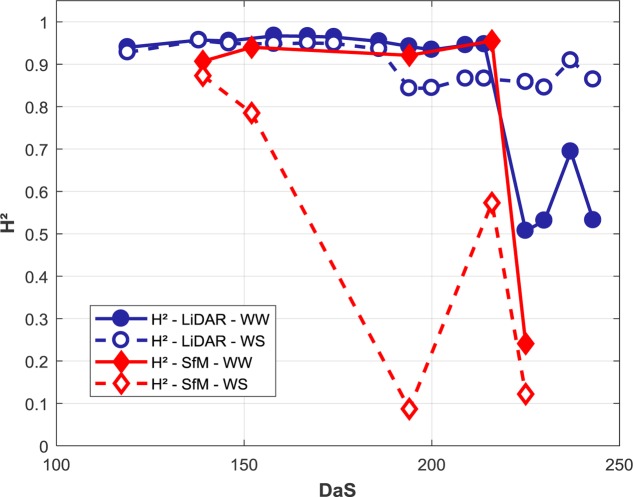
Heritability (*H*^2^) of plant height for different environments conditions and methods along the growth cycle. The two modalities (WW and WS) and plant height derived from LiDAR and structure from motion are individually presented.

The principles of height measurement are very different between the LiDAR and structure from motion: the structure from motion algorithm uses two different directions to build the dense cloud, limiting the penetration capacity because of possible occultation; conversely LiDAR uses only a single direction with much better penetration in the canopy. As a consequence, the z profiles are expected to be different between LiDAR and structure from motion. The impact of the *R*_p_ value used to define plant height from the dense cloud derived from structure from motion was thus further investigated on the 2076 couples of measurements. As expected, increasing the *R*_p_ value decreases the bias and thus RMSE with the reference LiDAR plant height (**Figure 7**). However, the decrease seems to be limited after *R*_p_ > 99%, reaching 8 cm difference for *R*_p_ = 99.99% Note that the 99.99% percentile corresponds to very few points in the dense point cloud since the cell of 0.5 m × 0.6 m contains around 1000 points. Increasing *R*_p_ reduces the variability of plant height between the 20 elementary cells within a microplot up to *R*_p_ = 99.9% (**Figure 8**). This simple sensibility analysis shows that best consistency with the LiDAR reference plant height is obtained for 99.5% < *R*_p_ < 99.99% with actually small improvement for *R*_p_ larger than 99.5%. This justifies a posteriori the *R*_p_ = 99.5% value used for plant height estimation from structure from motion.

### Plant Height as a Reliable Trait for Wheat Phenotyping

#### Broad Sense Heritability

The *H*^2^ quantifying the repeatability of the plant height trait estimation was computed as the ratio between the genotypic to the total variances (Holland et al., 2002). A linear mixed-effects statistical model was applied on each date to quantify the genetic variance. The ‘lm4’ R package applied to our experimental design (alpha design) was used here (Bates et al., 2014). The soil water holding capacity that was carefully documented was used as fixed effect in the model. We write the model (random terms underlined) as:

$$Y=\mu +S+\underset{-}{G}+\underset{-}{L}+\underset{-}{C}+\underset{-}{L:C}+\epsilon$$

Where *S* is the soil water holding capacity. *G* is the random effect of the genotypes. *L* and *C* are, respectively, the random lines and column effects in our alpha design plan and *L:C* is the random sub-block effect. μ is the intercept term (fixed) and ε the random residual error.

The plant height trait derived from the LiDAR shows a high *H*^2^ up to DaS 210 (**Figure 8**) for the WW modality. It drops dramatically at the end of the growth cycle in relation to lodging that was affecting differently the replicates. Conversely, the WS modality keeps relatively stable during the whole growth cycle because no lodging was observed. However, when the water stress starts to impact crop growth around DaS 180, a small decrease of the *H*^2^ is observed: residual environmental effects not accounted for by the alpha experimental plan and the soil water holding capacity were slightly degrading the *H*^2^ value.

The *H*^2^ values computed over the WW modality from structure from motion are close to those observed for the LiDAR, with, however, a slight degradation of the performances. Conversely, the *H*^2^ values computed on the WS modality from structure from motion show the smallest *H*^2^ values. On DaS 194, the *H*^2^ is low for the WS modality. A detailed inspection shows a noisy dense point cloud in the WS part of the field that impacted the height computation and thus *H*^2^. At this specific date and location, the phénomobile was operating during the UAV flights which induces artifacts and problems in the dense point cloud generation from structure from motion.

#### Plant Height as a Proxy of the Flowering Stage

**FIGURE 9:**
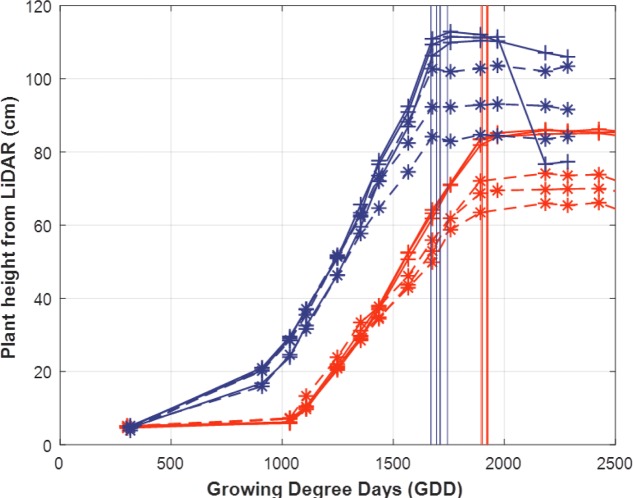
Dynamics of the LiDAR plant height for two genotypes (red and blue lines and symbols) with three replicates in the WW environment (‘+’ solid lines) and in the WS environment (‘^∗^’ dashed lines) the date when the maximum plant height is reached is indicated by the vertical line. Time is expressed in Growing Degree Days (GDD, °C.day).

Due to the reduced observation frequency of the UAV, flowering time was only assessed using the LiDAR plant height. The date when the maximum plant height is reached, *D*_max(PH)_, is considered as a proxy of the flowering stage. **Figure 9** shows that *D*_max(PH)_ is well identified based on the simple algorithm presented in the methods section. Further, it appears that *D*_max(PH)_ is little dependent on the environmental conditions: WW and WS modalities are very close and for the WS modality, there is no difference due to the soil water holding capacity although differences in *max(PH)* are observed.

**FIGURE 10:**
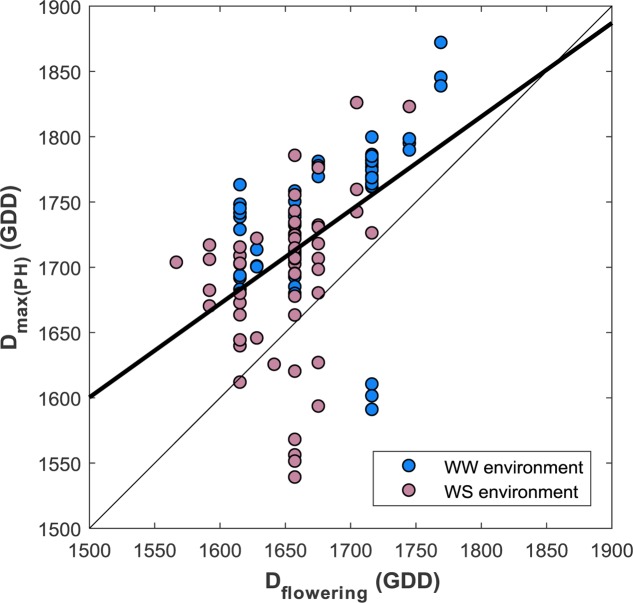
Comparison between the date expressed in Growing Degree Day (GDD) of the maximum plant height growth with the flowering date visually scored (expressed in GDD) (*n* = 114).

The flowering dates are well correlated with *D*_max(PH)_ (**Figure 10**) (*R*^2^ = 0.24, RMSE = 76, *D*_max(PH)_ = 0.7 D_flowering_ + 541). However, the best linear fit shows that the earlier genotypes reach the maximum plant height about 100 GDD after the flowering stage, which corresponds approximately to 7 days. The late genotypes show less differences, around 20 GDD corresponding to 1 or 2 days after flowering. *D*_max(PH)_ appears thus to be a reasonable proxy of the flowering stage considering that the accuracy of its visual scoring date is around 2–3 days. Nevertheless, some genotypes show significant differences from the main relationship as illustrated in **Figure 10**.

The heritability of *D*_max(PH)_ was very high, *H*^2^ = 0.96 and *H*^2^ = 0.88, respectively for the WW and WS modalities. This confirms the small influence of the environment for the genetic expression of this trait.

#### Relationship with Above Ground Biomass and Yield

**FIGURE 11:**
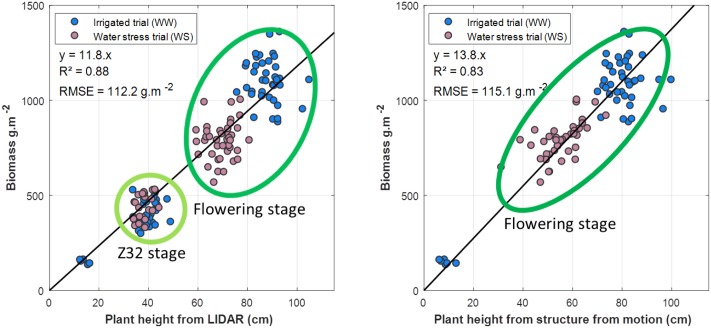
(left) Regression between plant height derived from LiDAR and Biomass (*n* = 139): (right) regression between plant height derived from structure from motion and Biomass (*n* = 86). Blue and red symbols correspond respectively to the WW and WS modalities. Unfortunately, no UAV acquisition was conducted for the Zadocks 32 stage. The Z32 and flowering stages are indicated by the corresponding green envelope of the points in the figures.

Correlations between plant height and biomass along the growing season are very strong (**Figure 11**) both for the LiDAR (*R*^2^ = 0.88, RMSE = 112.2 g/m^2^) and the structure from motion (*R*^2^ = 0.91, RMSE = 98.0 g/m^2^). These good relationships confirm observations by several authors (Yin et al., 2011; Bendig et al., 2014; Ota et al., 2015; Tilly et al., 2015). However, these correlations are mainly driven by the variability across stages along the growth cycle. For a given stage, little prediction power of the biomass is observed from plant height (**Figure 11**). The correlation at the flowering stage is relatively low (*R*^2^ = 0.5) for both methods. Other variables such as the basal area should be used to improve the correlations. Yield is poorly correlated with maximum plant height both when derived from LiDAR (*R*^2^ = 0.22, RMSE = 149.6 g.m^-2^) and structure from motion (*R*^2^ = 0.13, RMSE = 152.3 g.m^-2^). This is consistent with the poor correlation with biomass observed for a given growing stage, assuming that the harvest index varies within a small range.

## Discussion and Conclusion

Since crop surface is very rough, an important point addressed in this study was to propose a definition of plant height from the 3D point cloud retrieved from LiDAR or structure from motion techniques. The 99.5% percentile of the cumulated *z*-value was found to be optimal for comparison with ground ruler measurements while minimizing the spatial variability over each microplot. However, this definition will probably slightly depend on the canopy surface roughness. As a consequence, the 99.5% percentile used as a reference for wheat should be checked and possibly adapted for other crops as well as a function of the spatial resolution used. LiDAR measurements are based on a single source/view configuration allowing to penetrate into the canopy and reach the ground reference surface. Plant height could then be directly measured because of the availability of ground reference points within a microplot. Conversely, the penetration capacity of structure from motion methods based on the combination of distinct view directions from the UAV is limited because of possible occultation that will increase when the canopy closes. In these conditions, two strategies were compared: (1) either find ground reference points over the whole 3D dense point cloud and interpolate these points to get the digital terrain model; or (2) use and ancillary digital terrain model, that was in this study derived from real time kinetics GPS acquired during the sowing of the crop. The first approach might be limited in the case of a terrain presenting a complex topography when only few ground points are identified. Note that the ground control points could be used as ground level points if the distance to the ground is precisely known. Results show that both methods reach the same level of accuracy. For the two approaches investigated here to define the digital terrain model and extract the plant height of each microplot, the methods presented here were designed to process automatically the original imagery. This includes automatic and direct extraction of the microplots as well as of the digital terrain model from the dense cloud as opposed to earlier studies where plant height was derived from a crop surface model generated from the dense cloud (Bareth et al., 2016).

The comparison between plant height derived from LiDAR and structure from motion shows a very high consistency with strong correlation (*R*^2^≈ 0.98) and small RMSE values (RMSE = 8.4 cm). Most of the RMSE was explained by a significant bias, the plant height being underestimated. This may be partly due to the differences in the spatial resolution of the two systems (about 4 mm for LiDAR and 10 mm for UAV imagery) as well as in differences in canopy penetration capacity. However, plant height derived from structure from motion is systematically lower than that of the LiDAR. Our results further indicate that larger field of view with shorter focal lengths would generate more accurate 3D dense point clouds from structure from motion and thus plant height because of the increased disparity between the several view points. However, complementary study should investigate more deeply this effect as well as the impact of a degraded spatial resolution.

High *H^2^* (repeatability) of plant height was observed both for LiDAR and structure from motion. The water stress experiment over which the LiDAR and structure from motion techniques were evaluated shows that plant height is a very pertinent trait to characterize the impact of drought before flowering stage: plant height not only quantifies the magnitude of the stress, it allows also to date precisely when the stress started to impact plant growth if sufficiently frequent observations are available. In addition, the date when plant height reaches its maximum was demonstrated to be a reasonable proxy of the flowering date with, however, some slight variability between genotypes. The heritability of the *D*_max(PH)_ reached was very heritable since it was demonstrated to be very little dependent on the water stress experienced by the plants in this experiment. The phasing difference between the end of the vegetative growth period and the flowering date might be investigated by breeders as a new trait of interest. Finally, plant height provides obviously a very easy and convenient way to identify plant lodging either based on the temporal evolution of the microplot, or from the variance between the 20 elementary cells considered in each microplot. All these results make the plant height trait very interesting for plant breeders. However, very low correlation with total above ground biomass and yield were observed for a given date of observation while high correlations are found across stages. Additional variables should be used such as the basal area to get the biovolume to get a better proxy of the above ground biomass at harvest.

Plant height derived from UAV using structure from motion algorithms were demonstrated here to lead to similar degree of accuracy as compared to the LiDAR observations from the phénomobile. The affordability and flexibility of UAV platforms and the constant improvement of cameras (better, smaller, lighter, cheaper) will probably make UAVs the basic vehicle to be used for high-throughput field phenotyping of plant height. Further, the recent availability of centimetric knowledge of the camera position for each image based on real time kinetics techniques will ease the structure from motion processing while possibly limiting the number of ground control points to be set up in the field.

## Author Contributions

SJ manage the field platform. SM and MH design the flight plan. ST, BdS, FB, and AC manage the phénomobile acsuisition. MH pilot the UAV. GC develop some routines for the processing of the images from the UAV. ST develop some routines for the preprocessing of the LiDAR. The algorithm development were mainly accomplished by SM, with the advices and comment from FB, BdS, and DD. SM wrote the manuscript and FB made very significant revisions. All authors participated in the discussion.

## Conflict of Interest Statement

The authors declare that the research was conducted in the absence of any commercial or financial relationships that could be construed as a potential conflict of interest.

## Footnotes

1 www.pix4d.com

2 www.agisoft.com

## Abbreviations

DaS: day after sowing
*D*_flowering_: date of flowering
*D*_max(PH)_: date of maximum height
GDD: growth degree day
GSD: ground sampling distance
*H^2^*: broad sense heritability
LiDAR: light detection and ranging
RMSE: root mean square error
*R*_p_: rank percentile
UAV: unmanned aerial vehicle
WS: water stress modality
WW: well watered modality

## References

[B1] Bareth G. Bendig J. Tilly N. Hoffmeister D. Aasen H. Bolten A. (2016). A comparison of UAV- and TLS-derived plant height for crop monitoring: using polygon grids for the analysis of Crop Surface Models (CSMs). *Photogramm. Fernerkund. Geoinf.* 2016 85–94. 10.1127/pfg/2016/0289

[B2] Bates D. Mächler M. Bolker B. Walker S. (2014). Fitting linear mixed-effects models using lme4. arXiv:1406.5823

[B3] Bendig J. Bolten A. Bareth G. (2013). UAV-based imaging for multi-temporal, very high Resolution Crop Surface Models to monitor Crop Growth VariabilityMonitoring des Pflanzenwachstums mit Hilfe multitemporaler und hoch auflösender Oberflächenmodelle von Getreidebeständen auf Basis von Bildern aus UAV-Befliegungen. *Photogramm. Fernerkund. Geoinf.* 2013 551–562. 10.1127/1432-8364/2013/0200

[B4] Bendig J. Bolten A. Bennertz S. Broscheit J. Eichfuss S. Bareth G. (2014). Estimating biomass of barley using Crop Surface Models (CSMs) derived from UAV-based RGB imaging. *Remote Sens.* 6 10395–10412. 10.3390/rs61110395

[B5] Berry P. M. Sterling M. Baker C. J. Spink J. Sparkes D. L. (2003). A calibrated model of wheat lodging compared with field measurements. *Agric. For. Meteorol.* 119 167–180. 10.1016/S0168-1923(03)00139-4

[B6] Blonquist J. M. Norman J. M. Bugbee B. (2009). Automated measurement of canopy stomatal conductance based on infrared temperature. *Agric. For. Meteorol.* 149 2183–2197. 10.1016/j.agrformet.2009.10.003

[B7] Brocks S. Bendig J. Bareth G. (2016). Toward an automated low-cost three-dimensional crop surface monitoring system using oblique stereo imagery from consumer-grade smart cameras. *J. Appl. Remote Sens.* 10 046021–046021. 10.1117/1.JRS.10.046021

[B8] Chéné Y. Rousseau D. Lucidarme P. Bertheloot J. Caffier V. Morel P. (2012). On the use of depth camera for 3D phenotyping of entire plants. *Comput. Electron. Agric.* 82 122–127. 10.1016/j.compag.2011.12.007

[B9] Dandois J. P. Ellis E. C. (2013). High spatial resolution three-dimensional mapping of vegetation spectral dynamics using computer vision. *Remote Sens. Environ.* 136 259–276. 10.1016/j.rse.2013.04.005

[B10] Deery D. Jimenez-Berni J. Jones H. Sirault X. Furbank R. (2014). Proximal remote sensing buggies and potential applications for field-based phenotyping. *Agronomy* 4 349–379. 10.3390/agronomy4030349

[B11] Escolà A. Planas S. Rosell J. R. Pomar J. Camp F. Solanelles F. (2011). Performance of an ultrasonic ranging sensor in apple tree canopies. *Sensors* 11 2459–2477. 10.3390/s110302459 22163749 PMC3231637

[B12] Fraser R. H. Olthof I. Lantz T. C. Schmitt C. (2016). UAV photogrammetry for mapping vegetation in the low-Arctic. *Arctic Sci.* 2 79–102. 10.1139/as-2016-0008

[B13] Geipel J. Link J. Claupein W. (2014). Combined spectral and spatial modeling of corn yield based on aerial images and crop surface models acquired with an unmanned aircraft system. *Remote Sens.* 6 10335–10355. 10.3390/rs61110335

[B14] Gitelson A. A. (2004). Wide dynamic range vegetation index for remote quantification of biophysical characteristics of vegetation. *J. Plant Physiol.* 161 165–173. 10.1078/0176-1617-01176 15022830

[B15] Granshaw S. I. (1980). Bundle adjustment methods in engineering photogrammetry. *Photogramm. Rec.* 10 181–207. 10.1111/j.1477-9730.1980.tb00020.x

[B16] Grenzdörffer G. J. (2014). Crop height determination with UAS point clouds. *Int. Arch. Photogramm. Remote Sens. Spat. Inf. Sci.* 1 135–140. 10.5194/isprsarchives-XL-1-135-2014

[B17] Harwin S. Lucieer A. Osborn J. (2015). The impact of the calibration method on the accuracy of point clouds derived using unmanned aerial vehicle multi-view stereopsis. *Remote Sens.* 7 11933–11953. 10.3390/rs70911933

[B18] Hoffmeister D. Waldhoff G. Korres W. Curdt C. Bareth G. (2015). Crop height variability detection in a single field by multi-temporal terrestrial laser scanning. *Precis. Agric.* 17 296–312. 10.1007/s11119-015-9420-y

[B19] Holland J. B. Nyquist W. E. Cervantes-Martínez C. T. (2002). “Estimating and Interpreting heritability for plant breeding: an update,” in *Plant Breeding Reviews*, ed. Janick J. (Hoboken, NJ: John Wiley & Sons, Inc.), 9–112. 10.1002/9780470650202.ch2

[B20] Holman F. H. Riche A. B. Michalski A. Castle M. Wooster M. J. Hawkesford M. J. (2016a). High throughput field phenotyping of wheat plant height and growth rate in field plot trials using UAV based remote sensing. *Remote Sens.* 8:1031 10.3390/rs8121031

[B21] James M. R. Robson S. (2014). Mitigating systematic error in topographic models derived from UAV and ground-based image networks. *Earth Surf. Process. Landf.* 39 1413–1420. 10.1002/esp.3609

[B22] Khanna R. Moller M. Pfeifer J. Liebisch F. Walter A. Siegwart R. (2015). “Beyond point clouds - 3D mapping and field parameter measurements using UAVs,” in *Proceedings of the IEEE 20th Conference on Emerging Technologies & Factory Automation (ETFA)*, Luxembourg, 1–4. 10.1109/ETFA.2015.7301583

[B23] Lefsky M. A. Cohen W. B. Parker G. G. Harding D. J. (2002). Lidar remote sensing for ecosystem studies lidar, an emerging remote sensing technology that directly measures the three-dimensional distribution of plant canopies, can accurately estimate vegetation structural attributes and should be of particular interest to forest, landscape, and global ecologists. *BioScience* 52 19–30. 10.1641/0006-3568(2002)052[0019:LRSFES]2.0.CO;2

[B24] Lisein J. Pierrot-Deseilligny M. Bonnet S. Lejeune P. (2013). A photogrammetric workflow for the creation of a forest canopy height model from small unmanned aerial system imagery. *Forests* 4 922–944. 10.3390/f4040922

[B25] Llorens J. Gil E. Llop J. Escolà A. (2011). Ultrasonic and LIDAR sensors for electronic canopy characterization in vineyards: advances to improve pesticide application methods. *Sensors* 11 2177–2194. 10.3390/s110202177 22319405 PMC3274039

[B26] Lowe D. G. (2004). Distinctive image features from scale-invariant keypoints. *Int. J. Comput. Vis.* 60 91–110. 10.1023/B:VISI.0000029664.99615.94

[B27] Mallet C. Bretar F. (2009). Full-waveform topographic lidar: state-of-the-art. *ISPRS J. Photogramm. Remote Sens.* 64 1–16. 10.1016/j.isprsjprs.2008.09.007

[B28] Moras J. Cherfaoui V. Bonnifait P. (2010). “A lidar perception scheme for intelligent vehicle navigation,” in *Proceedings of the 11th International Conference on Control, Automation, Robotics and Vision*, Singapore, 1809–1814. 10.1109/ICARCV.2010.5707962

[B29] Neckar P. Adamek M. (2011). Software and hardware specification for area segmentation with laser scanner SICK LMS 400. *J. Syst. Appl. Eng. Dev.* 5 674–681.

[B30] Ota T. Ogawa M. Shimizu K. Kajisa T. Mizoue N. Yoshida S. (2015). Aboveground biomass estimation using structure from motion approach with aerial photographs in a seasonal tropical forest. *Forests* 6 3882–3898. 10.3390/f6113882

[B31] Otsu N. (1979). A threshold selection method from gray-level histograms. *IEEE Trans. Syst. Man Cybern.* 9 62–66. 10.1109/TSMC.1979.4310076

[B32] Owen S. J. (1992). *An Implementation of Natural Neighbor Interpolation in Three Dimensions.* Masters thesis, Brigham Young University, Provo, UT.

[B33] Rawson H. Evans L. (1971). The contribution of stem reserves to grain development in a range of wheat cultivars of different height. *Aust. J. Agric. Res.* 22 851 10.1071/AR9710851

[B34] Remondino F. El-Hakim S. (2006). *Image-based 3D Modeling: A Review.* Available at: http://nparc.cisti-icist.nrc-cnrc.gc.ca/npsi/ctrl?action=rtdoc&an=8913373 [accessed March 22, 2016].

[B35] Remondino F. Grazia M. Nocerino E. Menna F. Nex F. (2014). State of the art in high density image matching. *Photogramm. Rec.* 29 144–166. 10.1111/phor.12063

[B36] Rusu R. B. Marton Z. C. Blodow N. Dolha M. Beetz M. (2008). Towards 3D point cloud based object maps for household environments. *Rob. Auton. Syst.* 56 927–941. 10.1016/j.robot.2008.08.005

[B37] Schima R. Mollenhauer H. Grenzdörffer G. Merbach I. Lausch A. Dietrich P. (2016). Imagine all the plants: evaluation of a light-field camera for on-site crop growth monitoring. *Remote Sens.* 8:823 10.3390/rs8100823

[B38] Seber G. A. F. (ed.) (1984). “Multivariate distributions,” in *Multivariate Observations*, (Hoboken, NJ: John Wiley & Sons, Inc.), 17–58. 10.1002/9780470316641.ch2

[B39] Smith M. W. Carrivick J. L. Quincey D. J. (2015). Structure from motion photogrammetry in physical geography. *Prog. Phys. Geogr.* 40 247–275. 10.1177/0309133315615805 27636005

[B40] Snavely N. Seitz S. M. Szeliski R. (2006). *Photo Tourism: Exploring Photo Collections in 3D. in ACM Transactions on Graphics*, 835–846. Available at: http://dl.acm.org/citation.cfm?id=1141964 [accessed January 3, 2017].

[B41] Tilly N. Aasen H. Bareth G. (2015). Fusion of plant height and vegetation indices for the estimation of barley biomass. *Remote Sens.* 7 11449–11480. 10.3390/rs70911449

[B42] Triggs B. McLauchlan P. F. Hartley R. I. Fitzgibbon A. W. (1999). “Bundle adjustment—a modern synthesis,” in *Proceedings of the International workshop on Vision Algorithms*, eds Triggs B. Zisserman A. Szeliski R. (Berlin: Springer), 298–372.

[B43] Tumbo S. D. Salyani M. Whitney J. D. Wheaton T. A. Miller W. M. (2002). Investigation of laser and ultrasonic ranging sensors for measurements of citrus canopy volume. *Appl. Eng. Agric.* 18 367–372. 10.13031/2013.8587

[B44] Turner D. Lucieer A. Wallace L. (2014). Direct georeferencing of ultrahigh-resolution UAV imagery. *IEEE Trans. Geosci. Remote Sens.* 52 2738–2745. 10.1109/TGRS.2013.2265295

[B45] Turner P. Tubana B. Girma K. Holtz S. Kanke Y. Lawles K. (2007). *Indirect Measurement of Crop Plant Height.* Stillwater, OK: Oklahoma State University.

[B46] van der Voort D. (2016). *Exploring the Usability of Unmanned Aerial Vehicles for Non-Destructive Phenotyping of Small-Scale Maize Breeding Trials.* Wageningen: Wageningen University and Research Centre.

[B47] Vautherin J. Rutishauser S. Schneider-Zapp K. Choi H. F. Chovancova V. Glass A. (2016). Photogrammetric accuracy and modeling of rolling shutter cameras. *ISPRS Ann. Photogramm. Remote Sens. Spat. Inf. Sci.* 3 139–146. 10.5194/isprs-annals-III-3-139-2016

[B48] Virlet N. Sabermanesh K. Sadeghi-Tehran P. Hawkesford M. J. (2016). Field Scanalyzer: an automated robotic field phenotyping platform for detailed crop monitoring. *Funct. Plant Biol* 44 143–153. 10.1071/FP16163 32480553

[B49] Yin X. McClure M. A. Jaja N. Tyler D. D. Hayes R. M. (2011). In-season prediction of corn yield using plant height under major production systems. *Agron. J.* 103:923 10.2134/agronj2010.0450

[B50] Zhang L. Grift T. E. (2012). A LIDAR-based crop height measurement system for *Miscanthus giganteus*. *Comput. Electron. Agric.* 85 70–76. 10.1016/j.compag.2012.04.001

